# The Inflammatory Bowel Disease Knowledge Inventory Device 2 (IBD-KID2) is an effective tool for measuring disease-specific knowledge in Chinese patients

**DOI:** 10.1371/journal.pone.0321036

**Published:** 2025-04-01

**Authors:** HaiQun Huang, Ping Li, Angharad Vernon-Roberts, Andrew S. Day, BaiLing Liu, ZhaoRu Wu, YuLing Liu, QiaoRu Ye, He Wang

**Affiliations:** 1 Department of Gastroenterology, Guangzhou First People’s Hospital, Guangzhou, Guangdong, China; 2 University of Otago Christchurch, Department of Pediatrics, Christchurch, New Zealand; University of Diyala College of Medicine, UNITED STATES OF AMERICA

## Abstract

**Background:**

The Inflammatory Bowel Disease Knowledge Inventory Device 2 (IBD-KID2) is a specialized tool designed to evaluate disease-specific knowledge in patients with inflammatory bowel disease. The aim of this study was to develop a Chinese version of IBD-KID2 and to test the reliability and validity of this tool in Chinese patients with IBD.

**Methods:**

A Chinese version of IBD-KID2 was developed through initial cultural relevance/comprehension review and adaptation using content validity index for individual items (I-CVI, level > 0.78 acceptable) and the scale overall (S-CVI, level > 0.8 acceptable). A standardized approach was used to translate IBD-KID2 to Chinese, with the final tool being 15 items long and scored as one point for each correct answer (maximum score of 15). Tool validity was evaluated in a convenience sample of patients with IBD. External reliability was evaluated using test-retest analysis in a participant subset two weeks after baseline completion and internal reliability evaluated using cohort scores (Cronbach’s alpha, Cronbach’s α).

**Results:**

Following expert review for cultural relevance/comprehension the original IBD-KID2 scored > 0.78 I-CVI and > 0.9 for the S-CVI, and the tool was then translated. Ninety-six participants with IBD completed the Chinese IBD-KID2; 68 (71%) were male, eight (8%) aged < 18 years, and 63 (66%) had Crohn’s disease. The mean IBD-KID2 score of the cohort was 9.2 (±3.2, range 3-14). Scores decreased with age (p = 0.012) and increased with higher levels of education (p < 0.001). The retest reliability in a subset of 30 patients showed a correlation of 0.89 (P < 0.001), with no difference between the two time points (mean difference 0.4, = 0.16). The tool had high internal consistency with a Cronbach’s α coefficient of 0.8.

**Conclusion:**

The Chinese version of the IBD-KID2 demonstrated satisfactory reliability and validity, making it a robust instrument for evaluating disease-specific knowledge in individuals with IBD.

## Introduction

Inflammatory bowel disease (IBD) is characterized as an incurable gastrointestinal inflammatory condition with an unknown cause, often manifesting with a progressive or relapsing-remitting course [1,2]. The two main sub-types of IBD are Crohn’s Disease (CD) and ulcerative colitis (UC). Symptoms of IBD can differ according to the area involved, disease severity, and specific manifestations. Symptoms can encompass pain, fatigue, abdominal pain, anorexia, urgency, and bloody diarrhea [3]. Ongoing inflammation can lead to disease progression and adverse outcomes such as abdominal abscesses, fistulas, strictures, and intestinal obstructions. Colitis is also associated with an increased risk of colorectal carcinoma [1].

Enhancing knowledge about IBD offers numerous benefits, including improved coping and self-management skills, greater adherence to treatment, heightened treatment effectiveness, and reduced healthcare costs [4–8]. Individuals with IBD have been shown to have varying levels of knowledge [5,9–11], and knowledge gaps among adolescents with IBD have included medications, tests, nutrition, growth, and the detrimental effect of smoking [6,10,12,13]. In China, research on the disease knowledge levels of patients with IBD has relied on non-standardized questionnaires created by individual research teams [11,14,15]. These non-standardized questionnaires have three main issues. First, the content of these non-standardized questionnaires is not specifically designed to assess the knowledge level of IBD patients. As a result, they may fail to accurately capture the nuances of patients’ understanding of their condition. Second, the development of these non-standardized questionnaires did not follow a rigorous questionnaire development procedure. This lack of structure can lead to inconsistencies and inaccuracies in the assessment process. Third, these non-standardized questionnaires were not subjected to reliability and validity testing. Access to a validated, reliable and standardized tool would enable comparisons and consistency.

In 2013, Haaland et al [12] developed and validated the Inflammatory Bowel Disease–Knowledge Inventory Device (IBD-KID) as a 23-item tool to measure disease-specific knowledge among children with IBD. This was subsequently refined and revalidated as IBD-KID2 following item-response analysis to reduce the number of questions to 15 items [16,17]. The IBD-KID2 was developed through a process of questionnaire development, questionnaire testing, and performance assessment, and multiple studies have confirmed that this questionnaire has good reliability and validity. The questionnaire encompasses knowledge areas such as general IBD awareness, treatment options, lifestyle considerations, and nutritional aspects. Scoring is based on the number of correct answers given, with a maximum of 15 points reflecting a higher level of disease-related knowledge.

IBD-KID2 has been validated in pediatric [10,17] and adult [18] English-speaking populations. In recent years, it has been translated into Italian [19], Arabic [20], Polish [21], and Korean [22], demonstrating robust reliability and validity, with many additional language studies underway. However, a suitable version has not yet been developed to enable the use of IBD-KID2 in China. Consequently, the objectives of this study were to develop and validate a Chinese language version of the tool with the aim of verifying the reliability and validity of the tool among Chinese patients with IBD.

## Methods

### IBD-KID2 assessment tool

The IBD-KID2 is a self-administered questionnaire designed to assess knowledge of IBD, comprising 15 items with six multiple-choice questions and nine true/false questions. Participants receive one point for each correct response, with a possible maximum score of 15.

### Study procedure

#### Cultural adaptation.

Three Gastroenterology experts were invited to assess the English version of IBD-KID2 and rate it for cultural and language relevance and comprehension, following a previously used and validated methodology [19]. Using the content validity index (CVI) process, experts were asked to provide a rating (0-10 Likert scale) for cultural/language relevance and comprehension for each item [23,24]. These scores were then combined to represent the proportion of the maximum possible score from all experts for individual items (Individual CVI, I-CVI), with each item needing to have an I-CVI > 0.78 to be included in the translation. For the overall tool the combined scale CVI (S-CVI) was required to be > 0.8 to be acceptable for translation.

The inclusion criteria for the experts were: (1) holding a senior professional title or above; (2) possessing an undergraduate degree or higher; (3) and having at least 10 years of experience in Gastroenterology.

#### Translation and back-translation of the IBD-KID2.

The English version of the IBD-KID2 was translated into Chinese using the ‘forward-backward’ approach. For the translation phase, two researchers proficient in both Chinese and English initially translated the IBD-KID2 tool. Any disagreements between the two preliminary translations were discussed and resolved with the input of a third researcher. Following this, two experts conducted language adaptation and further modifications on the translated drafts, incorporating their expert suggestions to create a preliminary draft of the IBD-KID2. In the back-translation phase, a bilingual expert and a medical English expert, both of whom were unfamiliar with the original scale, were invited to back-translate the preliminary draft to English. The back-translated questionnaire was then sent to the original English-speaking authors for review and revision to ensure that the meaning and intention of items had not been changed during the translation process. Based on the revision suggestions and expert discussions between the English and Chinese speaking research teams, additional modifications were made. Finally, a comparative analysis between the back-translated version and the original scale was conducted to further refine the content of the scale, thereby confirming the final Chinese version of the IBD-KID2. The Chinese version of IBD-KID2 can be found in S1 File. The back-translated IBD-KID2 can be found in S2 File.

### Validity testing study

#### Population.

Inclusion criteria for participants were as follows: (1) being at least 8 years old; (2) having a diagnosis of IBD based on established diagnostic standards; (3) having the ability to communicate verbally and understand the study requirements; and (4) willingly participating in the study after providing informed consent.

Exclusion criteria included: (1) patients with hearing impairments or communication difficulties; (2) patients with psychiatric conditions or those who were non-cooperative

#### Study centre.

The study enrolled adults and children with IBD admitted to the Department of Gastroenterology at Guangzhou First People’s Hospital in China between October 2023 and April 2024. All participants provided written informed consent, with minors obtaining consent signed by their parents or guardians. This trial has passed the ethical review by the hospital’s ethics committee (K-2023-099-01) and has been registered in the national medical research registration and filing system (MR-44-24-001716).

#### Pre-survey assessment by patients with IBD.

For pre-survey testing a convenience sampling was used to select a group of patients with IBD to read IBD-KID2 and provide feedback on whether the questionnaire contained ambiguous language or unintelligible items. Prior to completing the questionnaire, patients were informed of the purpose and significance of the study.

#### Main validity study.

**Fig 1:**
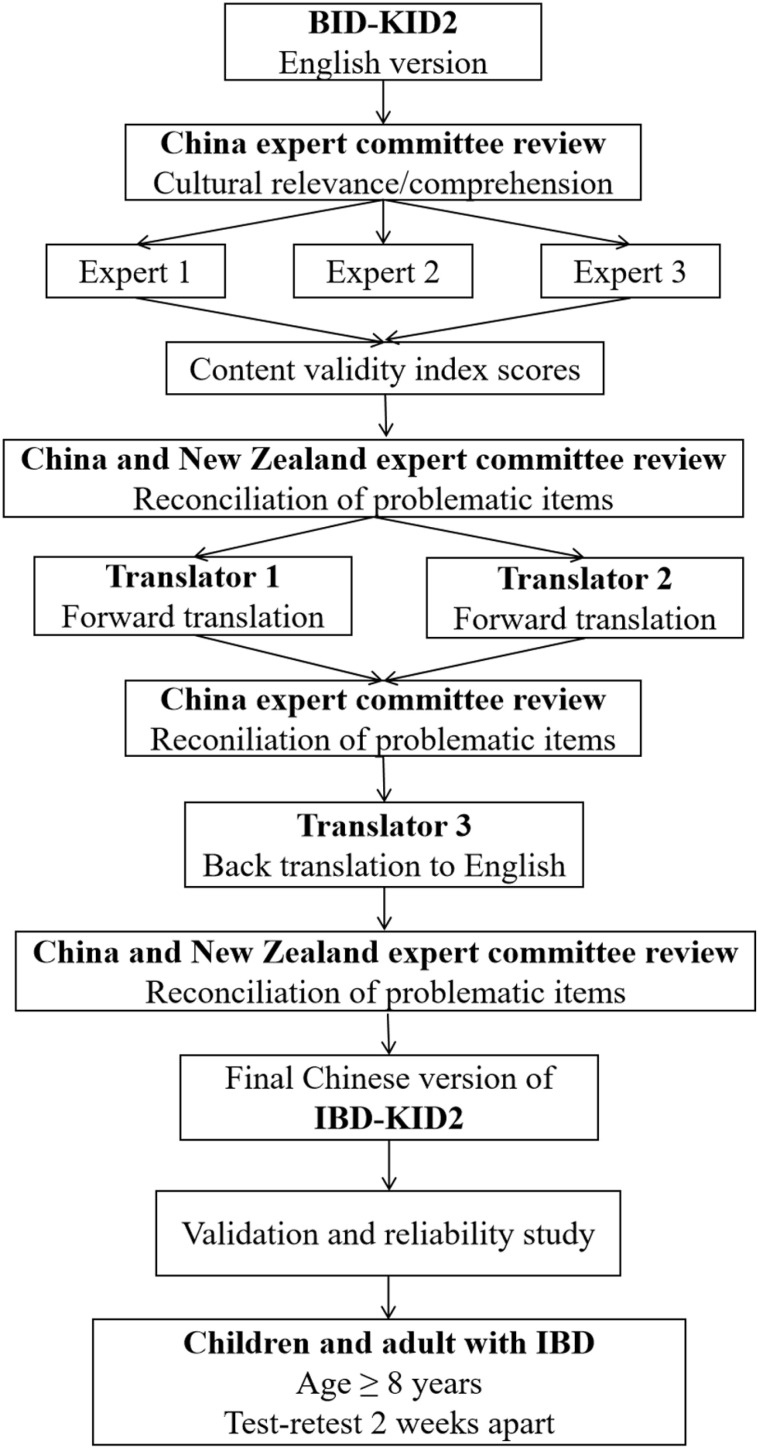
A flowchart of the methodology in this study.

For the full validity testing, a cohort of patients were recruited. All initially completed a baseline demographic survey to collect information on their sex, age, marital status, education level, occupation, diagnosis, disease duration, family history, place of residence, monthly household income, ethnicity, and religious belief. All participants were then asked to complete IBD-KID2. A sub-set of patients were asked to repeat IBD-KID2 two weeks later to assess test-retest reliability. The methodological flowchart is detailed in Fig 1.

### Statistical analysis

Continuous data were presented as means and standard deviations, whereas categorical data were depicted through counts and percentages. The analysis between IBD-KID2 scores and independent variables employed analysis of variance and Pearson correlation analysis.

External reliability was assessed using test-retest analysis, with IBD-KID2 completed two weeks after the baseline test to ascertain the consistency of its results. This timeframe was chosen to mitigate memory effects and the likelihood of participants acquiring new IBD knowledge during their patient journey. Scores between baseline and repeat were assessed using paired *t*-tests and intraclass correlation coefficient (ICC, reported as R), with test-retest ICCs considered to be showing good reproducibility if the value was ≥ 0.70.

Internal reliability was assessed using Cronbach’s α coefficient, and Spearman-Brown split-half reliability coefficient; carried out after assessment tools have been shortened in length. Cronbach’s Reliability (CR) is a measure of the internal consistency or reliability of a psychometric test.

For individual item analysis an initial Kaiser-Meyer-Olkin (KMO) test and Bartlett’s test of sphericity were carried out to determine suitability for factor analysis. Individual item analysis was carried out using both the extreme value method and correlation analysis method.

Overall, a *P*-value <  0.05 was considered statistically significant and data analysis was conducted using SPSS 26.0 and AMOS 26.0 software (IBM Corp, Armonk, New York, US).

## Results

### Content validity

Three experts completed the cultural/language review. The experts had working experiences ranging from 10 to 20 years, with one holding a doctoral degree, two with master’s degrees, and two with bachelor’s degrees. Based on the evaluation scores from three experts, the I-CVI ranged from 0.8 to 1.0, and the S-CVI was 0.93. These scores indicated that the cultural/language comprehension and relevance to Chinese patients with IBD was high and that no items required review or removal prior to translation.

### Pre-survey

Twenty-five participants with IBD completed the pre-survey asking them about the structure and content of IBD-KID2. Eight participants suggested clarifying what “steroids” are in Item 12 (“If there are side effects after taking steroids, one should immediately stop taking steroids.”). After discussion with the expert committee, no changes was made to item 12 in order to accurately assess the knowledge level of IBD patients. After completing the questionnaire, we provided an explanation of steroids for patients raising the question.

### Validation study

One hundred questionnaires were distributed for the validity study with 96 questionnaires returned, resulting in a response rate of 96%. The majority of the participants were male (71%), 66% had CD, and eight (8%) were children (Table 1). Just 10% of the cases had a family history of IBD. The duration of disease varied from less than one year to over five years.

**Table 1:** Demographic and patient-specific details from the overall cohort.

Categorical variable	Group	Frequency *N* (%)
Gender	Male	68 (71)
	Female	28 (29)
Age	8–17 years old	8 (8)
	18–30 years old	36 (38)
	31–60 years old	42 (44)
	≥61 years old	10 (10)
Marital status	Single	48 (50)
	Married	47 (49)
	Divorced	0 (0)
	Widowed	1 (1)
Education level	Elementary/Middle School	25 (26)
	High school	28 (29)
	University	43 (45)
Occupation	Staff	15 (16)
	Farmer	18 (19)
	Self-employed	7 (7)
	Retired/Unemployed	16 (17)
	Student	20 (21)
	Other	20 (21)
Diagnosis	CD	63 (66)
	UC	33 (34)
Disease Duration	<1 years	28 (29)
	1–3years	32 (33)
	3–5years	8 (8)
	>5 years	28 (29)
Family History	Yes	10 (10)
	No	86 (90)
Place of Residence	Urban	64 (67)
	Rural	32 (33)
Monthly Household Income	<3000 ¥	8 (8)
	3000-5000 ¥	28 (29)
	5000-10000 ¥	36 (38)
	≥10000 ¥	24 (25)
Ethnicity	Han Chinese	95 (99)
	Others	1 (1)
Religious Belief	Yes	3 (3)
	No	93 (97)

CD: Crohn’s disease, UC: ulcerative colitis.

### Knowledge survey scores

The mean IBD-KID2 score of the overall cohort 9.2 (SD 3.2) with scores ranging from 3 to 14 (out of maximum 15), representing a mean percentage score of 61% (SD 21, total score range 20-93%). The cohort of children scored marginally higher than the adults, with a score mean difference (MD) of 2.3/15 (15%) (P =  0.09, CI -5 to 0.4).

An examination of the correlation between individual patient variables and the knowledge scores garnered from each survey showed that younger participants scored higher (R 0.26, P =  0.009), and those with a university education scored higher than those with a High School education (MD 2.4, P 0.02) or elementary education (MD 3.2, P =  0.001) (Table 2).

**Table 2:** Associations between patient-specific characteristics and knowledge survey scores.

Categorical variable	Group	Pearson relation	*P* value
Gender	Male	0.02	0.83
	Female		
Age	8–17 years old	−0.26	0.01
	18–30 years old		
	31–60 years old		
	≥61 years old		
Marital status	Single	−0.18	0.08
	Married		
	Divorced		
	Widowed		
Education level	Elementary/Middle School	0.37	<0.001
	High school		
	University		
Occupation	Staff	0.01	0.94
	Farmer		
	Self-employed		
	Retired/Unemployed		
	Student		
	Other		
Diagnosis	CD	−0.16	0.12
	UC		
Disease Duration	<1 years	0.11	0.29
	1–3years		
	3–5years		
	>5 years		
Family History	Yes	−0.07	0.50
	No		
Place of Residence	Urban	−0.16	0.12
	Rural		
Monthly Household Income	<3000 ¥	0.13	0.21
	3000-5000 ¥		
	5000-10000 ¥		
	≥10000 ¥		
Ethnicity	Han Chinese	−0.13	0.19
	Others		
Religious Belief	Yes	−0.17	0.10
	No		

CD: Crohn’s disease, UC: ulcerative colitis

### Answer patterns

**Fig 2:**
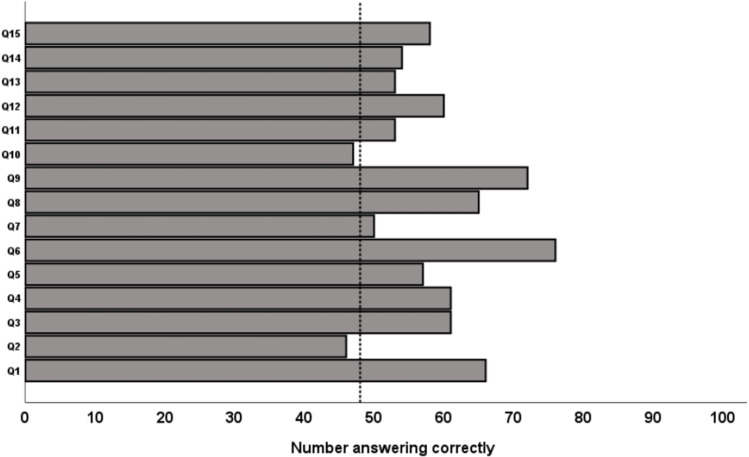
Patterns of disease knowledge according to number of participants giving correct answers to each individual Chinese IBD-KID2 item, with vertical line representing 50% of the overall cohort (48/96).

For 13 of the 15 IBD-KID2 items, more than 50% of the cohort answered correctly (Fig 2). The two items with less than 50% correct answers related to doctors and scientists knowing what causes IBD (Question 2) and knowing whether children will develop IBD if their parents have it (Question 10).

### Reliability analysis

#### External reliability.

Thirty participants completed IBD-KID2 after a two-week interval to assess reliability using test-retest analysis. The scores at baseline did not differ from the repeat test (mean difference 0.4, (SD 1.4), P = 0.16) and showed strong correlation between scores (R 0.9, P=<0.001).

#### Internal reliability.

The analysis of scores for each Chinese IBD-KID2 item showed that in the extreme groups of highest/lowest scorers, the CR values ranged between 3.6 and 9.8 (all *P* < 0.01). Correlation test outcomes demonstrated that the correlation coefficients between individual item scores and the total scale score varied from 0.37 to 0.74 (all *P* < 0.01). Notably, item 12 had a correlation coefficient of 0.37, while all other items showed coefficients above 0.4. Given that the scale comprises a modest total of 15 items and item 12’s *P* value is below 0.01, it has been tentatively retained (Table 3). The Chinese version of the IBD-KID2 had an overall Cronbach’s α of 0.80 and a Spearman-Brown split-half reliability of 0.80, indicating that the questionnaire has good internal reliability.

**Table 3:** Cronbach’s reliability values of IBD-KID2 and the correlation coefficients between item scores and total scores.

Item	High group (means ± SD)	Low group (means ± SD)	CR Values	Correlation coefficient between item scores and total scores
1	0.93 ± 0.05	0.43 ± 0.09	5.16	0.43
2	0.83 ± 0.07	0.29 ± 0.08	5.14	0.50
3	0.97 ± 0.05	0.26 ± 0.08	8.59	0.69
4	0.86 ± 0.07	0.34 ± 0.08	4.98	0.46
5	0.97 ± 0.03	0.46 ± 0.09	5.52	0.45
6	1.00 ± 0.00	0.37 ± 0.08	7.59	0.63
7	0.90 ± 0.06	0.23 ± 0.07	7.25	0.57
8	0.93 ± 0.05	0.29 ± 0.08	7.09	0.48
9	1.00 ± 0.00	0.49 ± 0.09	6.00	0.57
10	0.86 ± 0.07	0.09 ± 0.05	9.78	0.74
11	0.83 ± 0.07	0.29 ± 0.08	5.14	0.48
12	0.83 ± 0.07	0.43 ± 0.09	3.60	0.37
13	0.83 ± 0.07	0.31 ± 0.08	4.80	0.43
14	1.00 ± 0.00	0.63 ± 0.08	4.48	0.45
15	0.79 ± 0.08	0.17 ± 0.07	6.25	0.47

CR = Cronbach’s reliability, SD = standard deviation.

#### Construct validity.

The KMO value for the Chinese version of the IBD-KID2 was 0.78, indicating that it was suitable for exploratory factor analysis. Bartlett’s test of sphericity, with a chi-square value of 305 (*P* < 0.001), confirmed that the tested variables exhibit strong construct validity. Employing principal component analysis coupled with an oblique rotation method, factors were identified based on eigenvalues exceeding 1, extracting a total of four common factors with eigenvalues greater than 1. These factors account for a cumulative variance contribution rate of 53% (50%), signifying that the information from the study items was effectively captured. Each item has a loading value greater than 0.4 on its respective common factor. Factor 1 included seven items, Factor 2 included three items, Factor 3 included three items, and Factor 4 included four items. The 15 items have loading values ranging from 0.53 to 0.74 on their respective common factors, with communalities ranging from 0.41 to 0.89.

**Fig 3:**
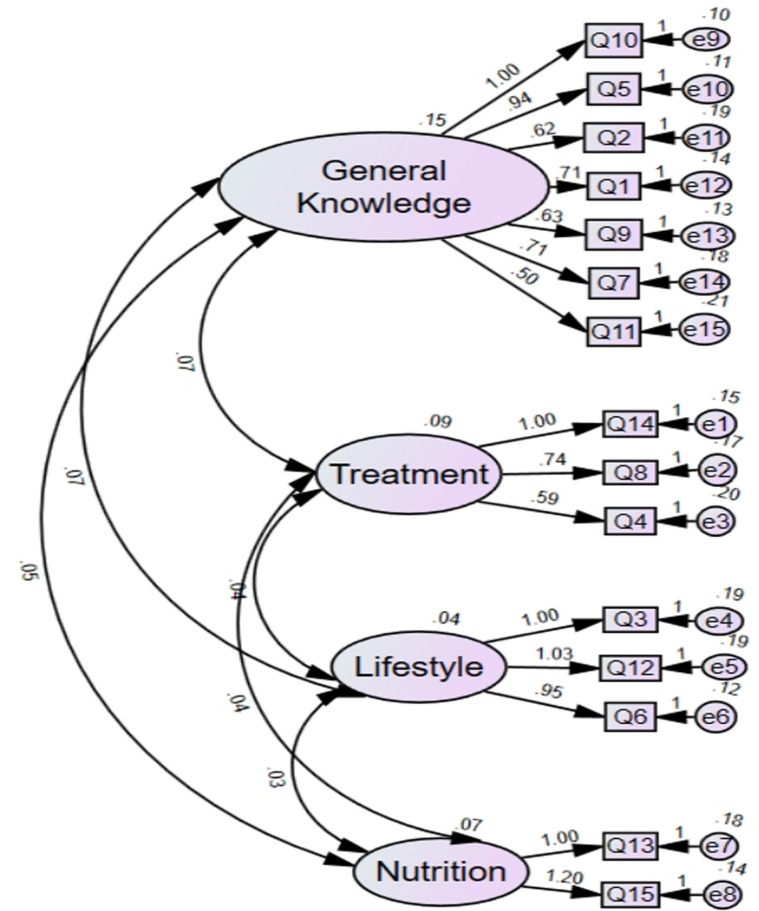
Confirmatory factor analysis structural equation modeling diagram for all Chinese IBD-KID2 items.

Drawing from the eigenvalues and the scope of content addressed by the original scale, the four factors have been designated as follows: Factor 1 for General Knowledge, Factor 2 for Treatment, Factor 3 for Lifestyle, and Factor 4 for Nutrition (Table 4, Fig 3). The confirmatory factor analysis yields the following fit indices: the χ²/ν ratio of 0.93, a Comparative Fit Index of 1.00, an Incremental Fit Index of 1.02, a Tucker-Lewis Index of 1.03, a Relative Fit Index of 0.7, a Normed Fit Index of 0.76, a Root Mean Square Error of Approximation of < 0.01, and a Root Mean Square Residual of 0.01.

**Table 4:** Results of the exploratory factor analysis for IBD-KID2.

Item	Factor Loadings				Communalities
	General Knowledge	Treatment	Lifestyle	Nutrition	
Question10.	**0.74**	0.26	0.42	0.21	0.66
Question 5	**0.74**	0.43	0.33	0.06	0.62
Question 2	**0.69**	0.08	0.07	0.13	0.56
Question 1	**0.62**	0.40	0.25	0.23	0.45
Question 9	**0.61**	0.31	0.28	0.07	0.41
Question 7	**0.54**	0.47	0.20	0.06	0.41
Question 11	**0.53**	0.07	0.06	0.32	0.47
Question 14	0.27	**0.70**	0.31	0.02	0.53
Question 8	0.32	**0.66**	0.19	0.20	0.61
Question 4	0.07	**0.61**	0.309	0.38	0.571
Question 3	0.23	0.08	**0.69**	0.30	0.54
Question 12	0.22	0.34	**0.68**	0.02	0.51
Question 6	0.47	0.08	**0.53**	0.09	0.43
Question 13	0.17	0.30	0.028	**0.81**	0.69
Question 15	0.30	0.07	0.318	**0.70**	0.59
Eigenvalues	4.17	1.41	1.18	1.17	
Cumulative variance contribution rate (%)	27.7	37.18	45.06	52.88	

## Discussion

The current study followed a standardized, comprehensive method of cross-cultural adaptation to translate IBD-KID2 from English to Chinese. Analysis showed that the Chinese IBD-KID2 is a valid and reliable assessment tool for adults and children with IBD that may now be implemented in the clinical and research setting.

Knowledge about IBD influences multiple dimensions of patients’ well-being, such as their quality of life [25], mental health [26], adherence to treatment [27–30], and coping mechanisms [29]. IBD management guidelines [31] emphasize that education and support in IBD knowledge are essential for evaluating the quality of IBD services. There is a growing focus on assessing and enhancing the knowledge levels of individuals with IBD, and a number of relevant tools are available. In 1999, Eaden et al [32] developed the CCKNOW questionnaire, comprising 24 items that evaluated five key areas: general knowledge, anatomy, medication, diet, and complications. Yoon et al [33] developed the IBD-KNOW knowledge questionnaire in 2019, which included 24 items covering anatomy, function, epidemiology, diet/lifestyle, common sense, medication, complications, surgery, and fertility aspects. Casellas et al. [34] developed the QUECOMIICAT questionnaire in 2019, consisting of 24 items addressing six areas: general concepts, clinical treatment, surgery, lifestyle habits, and social background. These assessment tools are relatively time-consuming in clinical practice and are not suitable for children, thus limiting their widespread application. Additionally, the CCKNOW tool was developed a number of years ago and may not reflect current disease understanding or approaches, such as use of biologic therapies. In contrast, the IBD-KID2 is shorter, simpler, and shown to be an effective, reliable, and scalable tool for assessing knowledge levels in many different populations. It is not only suitable for adults with IBD [18] but also for evaluating the knowledge levels of children with IBD and their parents [10]. Furthermore, it has been shown to be sensitive to change, such as following an educational activity [35].

In this current study, both age and level of education were identified as factors influencing disease-specific knowledge. In particular, older patients tended to have lower scores. This could be attributed to an age-related decline in memory or to the availability of education materials that may be less accepted by older participants such as material online or delivered via social media. Additionally, it was observed that individuals with higher education levels achieved higher scores than those with only primary or secondary education, which is consistent with the findings of numerous prior studies [18,33,36]. Previous research has demonstrated a correlation between sex and IBD knowledge scores, with females have superior disease-specific knowledge [36,37]. However, this correlation was not observed in the present study although the ratio of male to female participants was slightly skewed. Furthermore, while other studies have reported that patients with CD exhibit a higher level of IBD-specific knowledge compared to those with UC, the current study did not reveal any differences in knowledge scores between these two patient groups [17,38].

Reliability is an effective indicator for evaluating the stability of assessment tools such as IBD-KID2. In the current study the Cronbach’s α and split-half reliability were high, and comparable to results seen in similar studies, indicating that IBD-KID2 has good internal consistency reliability and can effectively reflect the level of disease-related knowledge among individuals with IBD. The test-retest method utilized to measure reliability of the Chinese version of the IBD-KID2 was higher than that of the English version, carried out among a cohort of children [17], indicating that the scale has good external stability reliability.

All but two items were answered correctly by more than half of the current study cohort. This differs to the patterns seen in other studies using the English version of IBD-KID2 among children [10,17] and adults [18], as well as in other translation studies [19–22]. Previous work among children of non-English speaking populations identified possible cultural differences in the IBD-KID2 items relating to food and nutrition with these scoring poorly [19,22]. However, this was not seen in the current cohort that was comprised predominantly of adults. Among an English-speaking adult cohort using IBD-KID2 the items regarding food were answered correctly by more than 50% of the study population [18], as in the current study. However, in that study of adults with IBD the item regarding growth was scored correctly by only one third of the participants, whereas more than 50% of the current study cohort scored this correctly. This highlights that knowledge scores may differ between cohorts for a variety of reasons that do not necessarily relate to cultural differences but may reflect independent factors such as prior patient education and utilization of knowledge sources.

## Strengths

This study represents a groundbreaking application of the IBD-KID2 questionnaire in China. The development and validation of the translated tool followed a standardized process as previously utilized. The completion and response rates have provided a robust basis for the study findings. As an inaugural endeavor in the Chinese context, this research not only broadens the applicability of the IBD-KID2 but also amplifies its influence, thereby laying a foundation for subsequent studies.

## Limitations

Firstly, the scope of this study was confined to a small cohort of children with IBD and it did not extend to evaluating the knowledge levels of their parents, even though prior studies have confirmed the IBD-KID2’s broader applicability among both children with IBD and their family members [10,35]. Consequently, future research should aim to include additional populations that include children with IBD and their parents, thereby further substantiating the efficacy, reliability, and generalization of IBD-KID2. Secondly, the current study did not undertake a comparative analysis of the Chinese version of IBD-KID2 with other scales, although this has been done previously in an English-speaking population of adults with IBD, showing preference of IBD-KID2 over CCKNOW [18]. At the same time, no adjustment for multiple comparisons was applied to Table 2 to avoid Type I errors. Lastly, the study drew on individuals cared for in a single hospital in China, which may limit the generalization of the findings. Therefore, subsequent studies should aim to diversify the sample, broaden the geographical scope, and amplify the sample size to enhance the robustness of the research outcomes.

## Conclusions

This study involved the translation and psychometric validation of a Chinese language version of the IBD-KID2 questionnaire. This short tool, covering four domains-general knowledge, treatment, lifestyle, and nutrition, is quick to complete and suitable for adults and children with IBD. It is thus convenient for clinical application and serves as a robust tool for the assessment of disease-specific knowledge. In conclusion, the Chinese version of the IBD-KID2 demonstrated good reliability and validity, confirming it to be an effective, reliable, and generalizable tool for evaluating the knowledge levels of individuals with IBD.

## Supporting information

S1 File Chinese version of the IBD-KID2. (DOCX)

S2 File The back-translated IBD-KID2. (DOCX)
