# Supplementary material for: The Inflammatory Bowel Disease Knowledge Inventory Device 2 (IBD-KID2) is an effective tool for measuring disease-specific knowledge in Chinese patients
Source: PLoS One. 2025 Apr 1;20(4):e0321036. doi: 10.1371/journal.pone.0321036 (PMC11960972; doi:10.1371/journal.pone.0321036)
Supplement: S1 File — (DOCX) [file pone.0321036.s001.docx]

炎症性肠病知识清单（IBD-KID2）

问卷说明：这不是一个考试，所以不要担心答错。请在每题您认为的答案前打勾✔。如果你不知道答案，就在“不知道”选项中打勾。请您自己回答这些问题。如果有需要，可以给您提供帮助，但我们需要你给出的答案。请不要在回答问题之前学习或寻求帮助得到正确的答案。如果您愿意完成该份炎症性肠病知识清单，请在同一时间完成所有的问题。不要完成一部分之后在另一天或另一段时间完成剩下的部分。非常感谢您花时间填写这份炎症性肠病知识清单。

1、从上到下，消化道的正确顺序是:（ ）

1. 口—胃—食道—大肠—小肠—肛门
2. 口—食道—胃—大肠—小肠—肛门
3. 口—食道—胃—小肠—大肠—肛门
4. 不知道

2、医生和科学家知晓炎症性肠病的病因。（ ）

A. 正确

B. 错误

C. 不知道

3、压力可以引起炎症性肠病的发作。（ ）

A. 正确

B. 错误

C. 不知道

4、您认为有可能需要进行肠镜检查的原因是：（ ）

A. 寻找病因

B. 切除部分病变肠道

C. 在肠道内用药

D. 不知道

5、炎症性肠病不仅仅影响肠道，还会影响其他器官。（ ）

A. 正确

B. 错误

C. 不知道

6、关于骨质疏松的描述，以下哪一项是正确的？（ ）

A. 男性或年轻女性不会得骨质疏松

B. 如果我喝大量牛奶，那我就不会得骨质疏松

C. 炎症性肠病可能引起骨质疏松

D. 不知道

7、缓解期的炎症性肠病会影响儿童/青少年的成长发育。（ ）

A. 正确

B. 错误

C. 不知道

8、治疗炎症性肠病的生物制剂药物是如何起作用的？（ ）

A. 减少感染的机会

B. 阻止化学物质或细胞引起炎症

C. 帮助身体吸收足够的营养物质

D. 不知道

9、如果炎症性肠病患者几个月没有症状，他们应该停止服用药物。（ ）

A. 正确

B. 错误

C. 不知道

10、如果父母双方都有炎症性肠病，那他们的孩子就会患炎症性肠病。（ ）

A. 正确

B. 错误

C. 不知道

11、关于补充和替代药品（如中草药）的描述以下哪一项是正确的？（ ）

A. 它们可能与处方药相互作用

B. 它们是天然的，所以没有副作用

C. 它们都可以与处方药安全使用

D. 不知道

1. 如果服用类固醇后有副作用，应立即停止服用。（ ）

A. 正确

B. 错误

C. 不知道

13、不吃某些食物（如牛奶）能阻止炎症性肠病加重。（ ）

A. 正确

B. 错误

C. 不知道

14、关于炎症性肠病手术的描述，以下哪一项是正确的？（ ）

A. 所有的炎症性肠病患者都需要手术

B. 手术对炎症性肠病患者没有帮助

C. 手术对部分炎症性肠病患者有帮助

D. 不知道

15、炎症性肠病患者吃了正确的食物就可以满足他们所有的营养需求。（ ）

A. 正确

B. 错误

C. 不知道
