# Supplementary material for: The Inflammatory Bowel Disease Knowledge Inventory Device 2 (IBD-KID2) is an effective tool for measuring disease-specific knowledge in Chinese patients
Source: PLoS One. 2025 Apr 1;20(4):e0321036. doi: 10.1371/journal.pone.0321036 (PMC11960972; doi:10.1371/journal.pone.0321036)
Supplement: S2 File — (DOCX) [file pone.0321036.s002.docx]

**List of inflammatory bowel disease knowledge**

Questionnaire instructions: Please read the following questions carefully and chose the answer you think is correct. ✔Check the letter before the option or fill in the brackets () with the letter. Please complete all questions in the questionnaire at the same time. This is not an exam, so there is no need to find the correct answer. Thank you for taking time to fill in this questionnaire!

1. From the top to bottom, the correct order of digestive tract is ( )
2. Oral cavity, stomach, esophagus, large intestine, small intestine, anus.
3. Oral cavity, esophagus, stomach, large intestine, small intestine, anus.
4. Oral cavity, esophagus, stomach, small intestine, large intestine, anus.

D. I don’t know.

1. Health workers know the cause of inflammatory bowel disease. ( )

A. Correct.

B. Wrong.

C. I don’t know.

1. Stress can lead to the onset of inflammatory bowel disease. ( )

A. Correct.

B. Wrong.

C. I don’t know.

1. The reason that you believe colonoscopy might be needed is ( ).

A. To look for the cause of illness.

B. To resect some part of the diseased intestine.

C. To administrate in the intestine.

D. I don’t know.

1. Inflammatory bowel disease affects not only the intestine, but also other organs. ( )

A. Correct.

B. Wrong.

C. I don’t know.

1. Regarding to osteoporosis, which of the following is correct? ( )

A. Men or young women will not suffer from osteoporosis.

B. If I drink a lot of milk, I will not suffer from osteoporosis.

C. The inflammatory bowel disease may lead to osteoporosis.

D. I don’t know.

7. Inflammatory bowel disease in remission can affect the growth and development of adolescents. ( )

A. Correct.

B. Wrong.

C. I don’t know.

8. How do the drugs work in treating inflammatory bowel disease?( )

A. By reducing the chance of infection.

B. By stopping chemicals or cells from causing inflammation.

C. By helping human body to absorb enough nutrients.

D. I don’t know.

1. If patients with inflammatory bowel disease have no symptoms for several months, they should stop taking the drug. ( )

A. Correct.

B. Wrong.

C. I don’t know.

10. If parents both have inflammatory bowel disease, their children will suffer from inflammatory bowel disease. ( )

A. Correct.

B. Wrong.

C. I don’t know.

11.Regarding the complementary and alternative medicines (e.g., Chinese herbs) , which of the following descriptions is correct ?( )

A. They may interact with prescription drugs.

B. They are natural, so they have no side effects.

C. They can all be used safely along with prescription drugs.

D. I don’t know.

1. If there are side effects after taking steroids, one should immediately stop taking steroids. ( )

A. Correct.

B. Wrong.

C. I don’t know.

1. Avoid eating some foods (such as milk) can prevent inflammatory bowel disease from deteriorating. ( )

A. Correct.

B. Wrong.

C. I don’t know.

1. Regarding the IBD surgery, which of the following descriptions is the correct? ( )

A. All patients with inflammatory bowel disease require surgery.

B. Surgery is not helpful for patients with inflammatory bowel disease.

C. Surgery is helpful for some patients with inflammatory bowel disease.

D. I don’t know.

1. By eating the right food, all nutritional needs of patients with inflammatory bowel disease can be meet. ( )

A. Correct.

B. Wrong.

C. I don’t know.
